# TOGETHER Project to Increase Understanding of the HIV Epidemic Among Sub-Saharan African Migrants: Protocol of Community-Based Participatory Mixed-Method Studies

**DOI:** 10.2196/resprot.5162

**Published:** 2016-03-17

**Authors:** Jasna Loos, Bea Vuylsteke, Lazare Manirankunda, Jessika Deblonde, Ilse Kint, Fiona Namanya, Katrien Fransen, Robert Colebunders, Marie Laga, Dorothy Adobea, Christiana Nöstlinger

**Affiliations:** ^1^ HIV and Sexual Health Unit Department of Public Health Institute of Tropical Medicine Antwerpen Belgium; ^2^ Epidemiology of Infectious Diseases Unit Department of Public Health and Surveillance Scientific Institute of Public Health Brussels Belgium; ^3^ HIV/STI clinic Department of Clinical Sciences Institute of Tropical Medicine Antwerpen Belgium; ^4^ AIDS Reference Laboratory Department of Clinical Sciences Institute of Tropical Medicine Antwerpen Belgium; ^5^ Department of Clinical Sciences Institute of Tropical Medicine Antwerpen Belgium; ^6^ Epidemiology for Global Health Institute Faculty of Medicine & Health Sciences University of Antwerp Antwerpen Belgium

**Keywords:** HIV, HIV prevention, sub-Saharan African migrants, HIV prevalence, HIV risk factors, mixed methods, community-based participatory research, oral fluid, electronic questionnaire, surveys and questionnaires

## Abstract

**Background:**

Sub-Saharan African Migrants (SAM) are the second largest group affected by HIV/AIDS in Belgium and the rest of Western Europe. Increasing evidence shows that, more than previously thought, SAM are acquiring HIV in their host countries. This calls for a renewed focus on primary prevention. Yet, knowledge on the magnitude of the HIV epidemic among SAM (HIV prevalence estimates and proportions of undiagnosed HIV infections) and underlying drivers are scarce and limit the development of such interventions.

**Objective:**

By applying a community-based participatory and mixed-methods approach, the TOGETHER project aims to deepen our understanding of HIV transmission dynamics, as well as inform future primary prevention interventions for this target group.

**Methods:**

The TOGETHER project consists of a cross-sectional study to assess HIV prevalence and risk factors among SAM visiting community settings in Antwerp city, Belgium, and links an anonymous electronic self-reported questionnaire to oral fluid samples. Three formative studies informed this method: (1) a social mapping of community settings using an adaptation of the PLACE method; (2) a multiple case study aiming to identify factors that increase risk and vulnerability for HIV infection by triangulating data from life history interviews, lifelines, and patient files; and (3) an acceptability and feasibility study of oral fluid sampling in community settings using participant observations.

**Results:**

Results have been obtained from 4 interlinked studies and will be described in future research.

**Conclusions:**

Combining empirically tested and innovative epidemiological and social science methods, this project provides the first HIV prevalence estimates for a representative sample of SAM residing in a West European city. By triangulating qualitative and quantitative insights, the project will generate an in-depth understanding of the factors that increase risk and vulnerability for HIV infection among SAM. Based on this knowledge, the project will identify priority subgroups within SAM communities and places for HIV prevention. Adopting a community-based participatory approach throughout the full research process should increase community ownership, investment, and mobilization for HIV prevention.

## Introduction

### Background

“Know your epidemic, know your response,” has become the directive of the Joint United Nations Programme on HIV and AIDS (UNAIDS) for intensifying HIV prevention [1]. Sub-Saharan African Migrants (SAM) are the second largest group affected by HIV/AIDS in Belgium [2,3]; however, knowledge on the population’s HIV prevalence and underlying factors that shape the HIV epidemic in SAM communities is scarce. This limits the development of targeted primary prevention interventions. Such interventions have gained importance, since recent evidence shows that increasing proportions of SAM acquire HIV in their European host countries [4-8]. To address this knowledge gap and improve primary prevention for SAM, we developed the TOGETHER Project, which started in January 2012. The project’s study protocol is presented in this paper.

### HIV in Belgium’s African Communities: “What Do We Know?”

In Belgium, 27% (n=230) of the newly reported HIV diagnoses in 2013 were in individuals of sub-Saharan African origin [3]. Since in 24% of all reported cases, data on the country of origin was missing, it is assumed that the overall number of new HIV diagnoses among SAM might be underestimated. Yet, it is clear that as communities of SAM are small (1.6% of the Belgian population), HIV disproportionately affects them.

Reported characteristics of newly diagnosed SAM in Belgium are in line with the generalized epidemic in sub-Saharan Africa. In 2013, the majority (64%) were women, heterosexual contact was the main transmission mode (89%), and most (78%) were diagnosed between 20 and 45 years [3]. Patients originated from 31 different countries, the largest groups coming from Cameroon (18%), Democratic Republic of the Congo (12%), and Guinea (9%) (personal communication Sasse, 2015). As in other European countries [2], HIV infections among SAM were usually diagnosed late. Of newly diagnosed SAM, 50% were late presenters, that is, their CD4 cell count was < 350/ml at the time of first diagnosis in Belgium [3]. According to CD4 cell decline simulations, it takes an estimated period of about four years from seroconversion for the CD4 count to reach 350 [9]. During this time, diagnostic opportunities may be missed. However, HIV diagnosis does not automatically translate into direct linkage to care among SAM [10,11]. Denial, coming to terms with diagnosis, not knowing where to go, feeling well and having no symptoms, HIV stigma and discrimination, and fear of medication prevent SAM from accessing care immediately after diagnosis [11].

Late diagnosis and delayed initiation of care not only affect disease prognosis [12], life expectancy [13], and health care costs [14-16], but also lead to an increased risk for onward HIV transmission, due to the prolonged period of unawareness of HIV status and continued high viral load of those not treated [17,18].

Belgian surveillance data show that 10.4% of all SAM diagnosed in 2013 report having acquired HIV in Belgium. Yet, this is based on the physician’s assessment at diagnosis and data are missing for 30% of cases [3]. Evidence from other EU countries suggests that this might be an underestimation. An Italian study among newly arrived immigrants first suggested that HIV infection had been more often acquired in the host country than previously estimated [6]. A study among newly diagnosed Africans in London specified that a quarter to a third of all HIV-positive Africans residing in the UK, and nearly half of HIV-positive African men who have sex with men (MSM), were likely to have acquired HIV in the UK [5]. Mathematical modeling applied to the UK’s national HIV-diagnosis data of heterosexuals born abroad suggests an increasing trend. While in 2004 an estimated 24% had acquired HIV after migration in the UK, this rose to 46% in 2010 [4], which accounts for an absolute increase of 16.5%. Preliminary results of applying the same mathematical model to Belgium’s national HIV surveillance data suggested that among patients newly diagnosed in 2011, 28% (IQR 24%-33%) of non-Belgium born heterosexuals and 39% (IQR 32%-47%) of non-Belgium born MSM could have acquired HIV in Belgium [7]. The close-knit sexual networks of SAM [19], combined with structural vulnerability related to the migration context [20,21], may contribute to an increased risk for HIV infection among SAM residing in Belgium.

### Knowledge Gaps for Effective HIV Prevention

HIV prevention comprises the continuum of primary prevention, promotion of HIV testing and counselling, and “positive health, dignity, and prevention,” which includes the prevention of HIV transmission. Yet, in Belgium [22] and its neighboring countries [23], prevention at the community level in the last decade focused mainly on the promotion of HIV testing and early linkage to care. Deeper understanding of SAM’s increased risk of being diagnosed late and the barriers to and facilitators of HIV-testing uptake [2,24-26] led to the development of multiple HIV-testing promotion strategies [14-16]. 

The prevention of new HIV infections, or primary prevention, among SAM has not been made priority because traditionally the HIV epidemic in the African diaspora in Europe was understood to be imported [27]. Evidence of SAM acquiring HIV after migration highlighted the need for tailored primary prevention interventions [4,5]. However, a number of gaps in in-depth understanding of transmission dynamics still exist. First, HIV prevalence estimates for a representative sample of the SAM communities in Europe are lacking, complicating HIV risk assessment and awareness raising in the communities. SAM have mostly formed subgroups in studies on HIV prevalence among other target groups, such as immigrant female sex workers [28], MSM [29], recently arrived migrants [6], and immigrants [30-34]. Only the MAYISHA II study, conducted in 2004 among 1359 black Africans in London, Luton, and the West-Midlands, provided HIV prevalence estimates of 14% [35]. Using a convenience sample of recruitment sites, this study did not include a representative sample, and subsequently, oversampling of HIV-positive individuals could not be excluded [4,5].

Second, estimates of the proportions of SAM with undiagnosed HIV are lacking. In Europe, one third of persons living with HIV are assumed to be unaware of their HIV status [21]. Some of the above-mentioned HIV prevalence studies provided indications that this might be higher among SAM; for example, MAYISHA II found that 66% of study participants with a positive test result did not report their HIV status on the questionnaire [35].

Third, previous research mainly focused on SAM’s individual knowledge, attitudes, and practices related to sexuality and HIV-preventive behavior, thus underestimating the social, cultural, religious, and migration-related contexts that increase vulnerability with respect to HIV [36]. Studies from the UK and the Netherlands underlined SAM’s preference for the heterosexual, monogamous standard [35]; yet, high number of partners (lifetime [37] and past year [30,35]), concurrent relationships, and having sex when traveling to home countries [30,34,37] were frequently reported, especially among men. This increased individual sexual risk behavior was shown to be linked to higher rates of sexually transmitted infections among SAM [34,37-39]. Several studies showed that SAM’s condom use was relatively high in comparison with the West-European population, but low considering their potential risk [40]. Africans were reported to believe that risk could be avoided by carefully choosing their sexual partners [40], to believe that condoms were associated with infidelity and reduced sexual pleasure, and to perceive condoms as inappropriate for long-term relationships [39,41-43]. Although these studies were useful to identify individual sexual risk patterns, they paid little attention to diversity among subgroups [36] and contextual factors influencing sexual behavior, and thus may be of limited use to develop and implement effective campaigns aiming to reduce HIV infection risk [44,45].

### SAM Communities in Antwerp City

Although small in numbers, SAM communities are characterized by a high degree of heterogeneity due to diverse ethnic and cultural backgrounds, migration patterns and residence statuses, educational and socioeconomic backgrounds, and religious beliefs [22,45]. Of the 175,000 SAM officially living in Belgium [46], about 17,400 (10%) reside in Antwerp city (according to data obtained from the City of Antwerp; email communication from 2012 May 23). These numbers include SAM who obtained Belgian nationality, second-generation Belgian-born children of SAM parents, registered migrants, and SAM whose residence procedure is pending. SAM of undocumented legal status are absent from these statistics. Almost half (47%) of SAM in Antwerp city originate from 3 countries: the Democratic Republic of the Congo (18.8%), Ghana (17.5%), and Nigeria (10.7%). Apart from these 3 main nationalities, 43 other nationalities are living in this area. In spite of their heterogeneity, SAM communities are fairly homogeneously organized. For many SAM, their nationality and/or ethnicity shape their social life. As new migrants, they depend on the social support of their compatriots to become settled [47]. As established migrants, they engage in social and cultural networks and use them to look for marriage and sex partners [30]. Although these ethnic networks can be described as tight, they are not ethnically segregated. Different ethnic groups mingle at commercial and social venues and events. To reach different communities with HIV prevention activities, partnerships with leaders (ie, of sociocultural or spiritual organizations and owners of commercial settings) are essential [48-50].

Many SAM live in socioeconomically vulnerable and legally unstable conditions. Together with prevalent HIV-related stigma and culturally grounded taboos on sexuality, this translates into little demand for HIV prevention [50,51] and increased risk for HIV acquisition [48].

## Methods

### Study Objectives

The TOGETHER study’s overall aim was to increase the communities’, researchers’, and policymakers’ in-depth understanding of the dynamics of the HIV epidemic among SAM, to improve primary prevention interventions. This translated into the following objectives:

1. To assess the HIV prevalence and proportion of undiagnosed HIV infections among SAM socializing in community settings in Antwerp city.
2. To identify individual, community-level, and structural risk factors for HIV infection among SAM.
3. To identify priority settings and groups for future primary HIV prevention interventions.
4. To increase community ownership, involvement, and mobilization for HIV prevention.
5. To develop policy recommendations to improve HIV prevention for the target group of SAM.
6. To assess the feasibility and acceptability of community-based participatory research on HIV prevalence in SAM communities and adopted research tools.

### Overall Study Design

**Figure 1:**
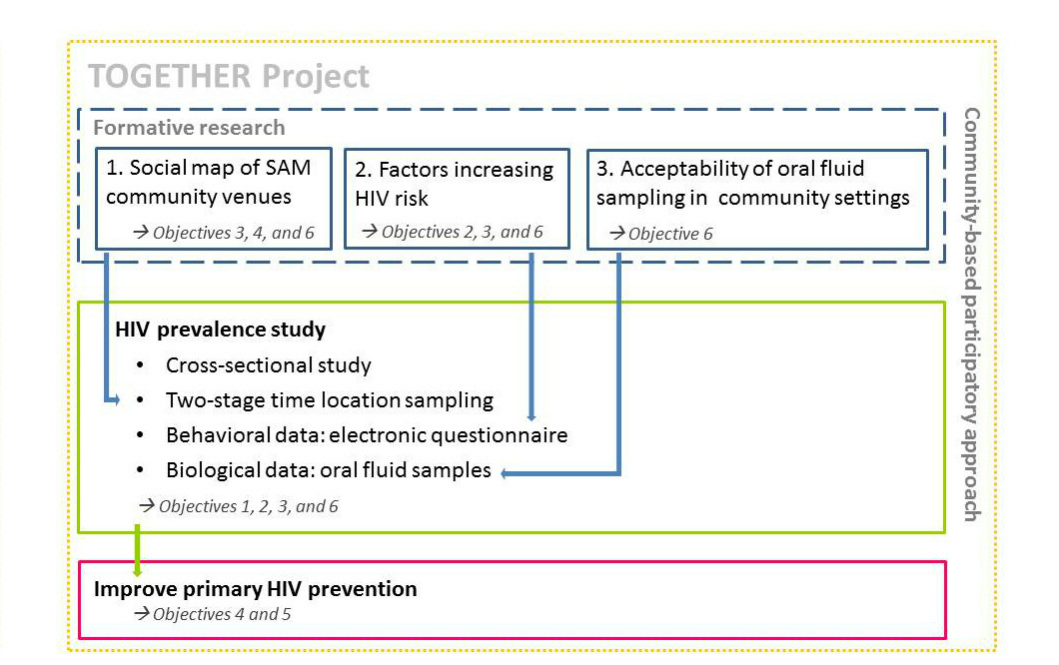
Outline of the TOGETHER project.

To meet these objectives, the TOGETHER Project applied mixed methods and a community-based participatory research approach (CBPR) [52]. The main study was a cross-sectional community-based bio-behavioral survey on HIV prevalence and HIV risk factors among SAM visiting community settings in Antwerp city (referred to as the “HIV prevalence study” in this article). To inform its design, 3 formative substudies were conducted. First, a social map of SAM community settings in Antwerp city was developed, applying an adaptation of the PLACE Method [53,54]. Second, factors that increase SAM’s risk of HIV infection were assessed using a multiple case study design. The third substudy assessed the acceptability and feasibility of using oral fluid collection devices for HIV testing in community venues through participatory observations, including informal interviews (see Figure 1).

For the different study components, separate study protocols were developed and ethical approval was obtained from the Institutional Review Board of the Institute of Tropical Medicine and the ethical committee of the University Hospital Antwerp.

### Community-Based Participatory Approach

To account for the heterogeneity of Antwerp’s SAM and to ensure that the study methods and tools were acceptable for all subgroups (objective 6), we chose a community-based participatory approach [52]. This methodology also allows for increasing community ownership, involvement, and mobilization for HIV prevention (objective 4) throughout the research process. In practice, community members are involved in all steps of the project, from conceptualization, to data collection, to development of new interventions and policy recommendations. We established collaborative partnerships both with research experts and communities [55], by engaging a team of community researchers (CRs) and setting up a community advisory board (CAB). The CAB hosted both leaders of African organizations and a multidisciplinary group of professionals. The CRs were 9 lay community members, who were trained at the start of the project and continuously coached throughout. To reflect the communities’ diversity, the CR team was diverse in its composition with respect to gender, age, origin, residence status, employment status, and HIV status. In line with the Greater Involvement of People living with HIV/ AIDS (GIPA) principles [56], we actively recruited SAM living with HIV for the CRs team.

### Formative Research

#### Formative Study 1: Social Mapping of Sub-Saharan African Community Venues in Antwerp City

This study ran from June 2012 until June 2013 and had the triple objective of (1) determining the sampling frame for the HIV prevalence study, (2) identifying priority settings for future HIV prevention, and (3) increasing communities’ ownership of HIV prevention.

To account for the heterogeneity of the SAM communities and ensure inclusion of hidden subpopulations, for example, SAM of undocumented status or MSM, we chose to employ the systematic approach of the PLACE Method (Prioritising Local AIDS Control Efforts) [53,54]. Guided by epidemiological theories, the PLACE Method has been widely used to monitor and improve AIDS prevention program coverage in areas where HIV transmission is most likely to occur, for example, by identifying venues where people meet sexual partners. It is a 5-step method adopted for surveillance studies, intervention design, programmatic up-scaling, and community mobilization [53,54,57-59]. We adapted the method to better meet our objectives by including steps 1 to 3, while replacing steps 4 and 5 with the HIV prevalence study (see below).

In step 1, “identifying a priority prevention area,” Antwerp city was selected based on demographic and epidemiological aspects: 22% of all SAM in Flanders live in Antwerp city [60] and, according to data available at the time, in 2010 43% of all newly diagnosed SAM in Flanders lived in Antwerp province. In step 2, “community informants” were interviewed, that is, adults who are knowledgeable about the community. Participants were asked where SAM socialize, and where they meet new sexual partners. We collected the locations of various publicly accessible meeting places such as bars, churches, shops, hair salons, asylum centers, a public library, parks, streets, squares, events, and festivities of African organizations. A convenience sample of community informants with different behavioral and sociodemographic characteristics, and of different professions, and community leaders of different origins, residing in different city districts, were interviewed to assure representativeness. After reaching saturation, duplications were removed from the compiled inventory of places and a consolidated list was generated. In step 3, all venues, areas, and events on the list were visited for a verification interview. Structured electronic questionnaires (SurveyToGo) were used to assess settings’ activities, characteristics of their visitors (origin, gender, etc.), busiest days and times, and existing HIV prevention programs, as well as to collect information on additional settings.

#### Formative Study 2: Assessing Factors that Increase Sub-Saharan African Migrants' Risk of HIV Infection: A Multiple Case Study

The second formative study contributed to the second overall project objective (ie, assessing individual, community-level, and structural risk factors). This study’s first phase took place between April 2013 and December 2013. The 3 specific objectives were: (1) informing the development of a structured questionnaire for the HIV prevalence study, (2) qualitatively contextualizing the findings of the HIV prevalence study, and (3) informing the development of future HIV prevention interventions. Study participants were SAM living with HIV, who were unique cases to retrospectively identify multi-level risk and vulnerability factors.

We conducted an empirical inquiry investigating a phenomenon within its real-life context, using multiple sources of evidence [61]. In our study, individual HIV positive SAM were the single object of a case. For each case, we triangulated findings from 3 data collection methods: life history interviews, timelines, and patient files. Per case, at least two life history interviews were conducted. Since chronology, sequencing of events, and context [62] are important to understand the setting in which HIV infection occurred, *timelines* were developed during these interviews. Timelines are visual depictions of an individual’s life events in chronological order that may include interpretations of these events [62]. We followed the timeline approach as described by Adrianson (2012), in which the drawing of the timeline is a collaborative effort shared by the interviewer and the interviewee, with the drawing forming the basis of the interview [63]. All interviews were conducted by a single interviewer (JL) who adopted an unstructured interview approach. This informal, open-ended, flexible, and free-flowing way of interviewing enabled participants to define the properties of the interview and direct the interview into areas that they saw as relevant [64]. The interview themes were the following: life story and events, migration (eg, pull and push factors, trajectory, immigration procedures), sexual and partner relationships, health-seeking behavior and coping styles and emotions (eg, effect of events on psychological well-being, coping with HIV, stigma and discrimination), social embeddedness (eg, social network, family structure and support, social exclusion) and livelihood (eg, financial situation, housing). When the natural flow of the first interview did not spontaneously generate sufficient data on these themes, a focused approach was taken in the second and eventual follow-up interviews. Data from the life history interviews were triangulated with data from the patient files. This reduced potential limitations of life history interviews (eg, subjective distortion), enriched the findings, and increased internal validity within the cases.

In the first study phase, a convenience sample of SAM living with HIV was recruited through physicians and nurses of the HIV clinic and facilitators of an HIV support group for SAM. To arrive at a representative sample of the patient population of sub-Saharan African origin, in the second phase, with a start date of April 2016, we switched to purposive sampling.

All participants in the first study phase were consenting adults who received their HIV diagnoses between 6 months and 10 years ago, who were assessed by health care providers as being psychologically stable and were followed up by a social nurse at the time of the interview. The latter was important to assure linkage to psychosocial care in case the interviews evoked emotional upheaval. The same rationale led to significant attention being paid to the informed consent procedure. Prior to the first interview, the study’s rationale, objectives, procedure, and confidentiality measures, and participants’ rights, benefits, and disadvantages were discussed extensively with the participant, before the informed consent form was signed. During this process, we asked for explicit approval to consult the participants’ patient files. For follow-up interviews, the procedure was repeated and verbal informed consent was obtained. As a token of appreciation, participants received an incentive of €25 for each interview.

To ensure participants’ anonymity and confidentiality, all data were coded and stored in a password-protected folder. All data were uploaded to NVivo 10 and a first *within-case analysis* was conducted. Data analysis adopted an inductive approach [65]. Triangulating the different data sources, the specific study questions were answered for each single case. Next, a cross-case analysis will be conducted at the end of the second study phase, to identify general factors that increase SAM’s vulnerability to HIV infection and facilitators of and barriers to behavior change.

#### Formative Study 3: Acceptability and Feasibility of Outreach HIV Testing Using Oral Fluid Collection Devices

For the HIV prevalence study, we opted for collecting oral fluid samples to determine HIV status, since reluctance toward blood taking is known to limit HIV testing uptake among SAM [66]. Although oral fluid collection devices had been used in comparable studies [30,35], none took place in Belgium; thus, their acceptability in SAM community settings was unknown. Therefore, an acceptability study was conducted between December 2012 and June 2013 within the framework of another HIV testing intervention named “swab2know.”

This intervention of the Institute of Tropical Medicine offered free oral fluid HIV tests (Oracol device, Malvern Medical Developments, Worcester, UK) in community settings of two target groups (MSM [67] and SAM) in Antwerp city. The HIV testing sessions for SAM were organized in collaboration with community leaders and included group counseling and testimony of an HIV-positive community member. If participants decided to test, they could choose to collect their result a week later via a secured website or face-to-face consultation at a low-threshold HIV testing center (ie, focusing on high-risk groups such as SAM). To assess the feasibility and acceptability of this intervention, including the specific sampling method for SAM, two social scientists (JL and FN) conducted participant observations [68] at 10 HIV testing sessions. Besides observation, informal interviews were conducted with testers, nontesters, and the intervention team. Field notes were coded using NVivo 10 using a data-driven code book and analyzed following inductive analysis principles [65].

### Community-Based Survey on HIV Prevalence and HIV Risk Factors Among Sam Visiting Community Venues in Antwerp City

The primary objective of this cross-sectional study, which ran from December 2013 to August 2014, was to determine HIV prevalence among SAM socializing in community settings in Antwerp city (objective 1 of the TOGETHER Project). Secondary objectives were: (1) Identifying the individual, community-level, and structural risk factors for HIV infection among SAM; and (2) Identifying priority settings for future HIV prevention interventions (project objectives 2 and 3, respectively).

### Sample Size

HIV prevalence was the primary outcome measure. The sample size was calculated using an anticipated HIV prevalence of 4%, a required precision of 2% for the 95% confidence intervals, and a cluster sampling design effect of 2. This resulted in a required sample size of 714 SAM.

### Sampling Method

A 2-stage time location sampling (TLS) was adopted. TLS takes advantage of the fact that some hard-to-reach populations tend to gather or congregate at certain types of locations [69]. The list of settings established in formative study 1 was the sampling frame, from which a 2-stage cluster probability sample was selected. At the first level of sampling, 51 clusters, or sites, were randomly selected from the list with a probability proportional-to-size. When a selected site was not available (eg, refusal of the bar owner, closure of the site, site moved out of study area), this was noted and the next site on the list was approached. The second level of sampling included the random selection of 14 study participants from each cluster.

To be eligible, potential study participants had to self-identify as belonging to the SAM communities, be 18 years or older, agree to answer the behavioral questionnaire, donate an oral fluid sample, and be willing and able to provide written informed consent. Prior participation in the prevalence study was an exclusion criterion.

### Measures

The study combined biological and behavioral measures. To measure the study’s primary outcome, HIV prevalence, oral fluid samples were collected and tested for HIV antibodies in the AIDS Reference Laboratory. These samples were linked through a unique code to an anonymous behavioral questionnaire. This instrument included questions on participants’ socio-demographic and economic background, migration and mobility background, health-seeking behavior, HIV-testing behavior, sexual and relational history (last year and lifetime), attitudes towards condom use, actual condom use, and level of assistance needed to complete the questionnaire. The structured electronic questionnaire was developed based on the findings of formative study 2, consultation of available questionnaires from comparable studies [39,42], and input from the CRs and CABs. It was refined after cognitive piloting with 12 participants and the pilot sessions (see below).

### Data Collection Procedures

Detailed study procedures were described in the Standard Operating Procedures (SOP) developed in collaboration with the CRs, CAB, and the AIDS reference laboratory. The SOP were refined after 2 pilots. At pre-arranged moments, a study team visited the selected sites and randomly selected 14 of the SAM present. When approaching potential participants, the CRs identified themselves and introduced the study’s objectives and methodology, stressing the anonymous and voluntary nature of participation. To avoid self-exclusion of HIV-positive individuals, they explicitly mentioned that everybody was invited to participate, regardless of HIV status. Interested individuals were invited to a quiet area in the setting, if available. After discussing and signing the informed consent form, participants were asked to complete a structured anonymous electronic questionnaire on a tablet through SurveyToGo. To build confidence and ensure data rigor, participants first received a short tutorial on how to use the tablet. Here, special attention was given to the demonstration of how anonymity was guaranteed. Questionnaires were available in French, English, and Dutch, which are reference languages for most SAM. The preferred interview method was self-completion; however, assistance was offered if needed. By ethnically matching the CRs to the settings, translation of questions to a local language was possible, if preferred. The CRs were trained to offer assistance with sensitivity and respect to confidentiality.

After completing the questionnaire, the CR demonstrated the procedure of oral fluid collection using the collection device. Next, participants were asked to self-collect the sample. The sample was then linked with the informed consent form, questionnaire, and a letter explaining how to collect the results through a *unique code* (no personal identifying information was requested). If they wished, participants could collect their HIV test result by calling the study nurse and providing their unique code, age, and country of origin. When results turned out to be HIV positive, participants were invited to the HIV testing center for confirmation testing, counselling, and linkage to care.

Finally, participants were asked to provide information on their frequency of *attending* the study settings and comparable settings. This allowed the calculation of a weighting factor (see “data analysis”). As a token of appreciation, participants received free condoms, an information brochure on HIV testing, and €5. Those who refused participation received condoms and the information brochure, but no financial compensation. Data on their characteristics and reasons for refusal were also collected, as well as the overall number of individuals present during the onsite data collection.

### Laboratory Procedures

Within 7 days of collecting the sample, the AIDS reference laboratory of the Institute of Tropical Medicine performed the analysis according to a validated algorithm using oral fluid specimens [70]. First, samples were tested with a Genscreen HIV ½ v2 (BioRad). If reactive, a second HIV ELISA test, Vironostika HIV Ag/Ab (BioMérieux) was performed. Only participants with 2 reactive test results were considered to be HIV-infected. For all negative samples, the quality of the oral fluid samples was measured using an IgG ELISA quantification kit (Human Total IgG ELISA, Immunology Consultants Laboratory, Inc, Cat No: E-80G). Samples were considered valid for analysis and results of the HIV test were only reported when sufficient IgG was present in the sample. All other samples were considered nonvalid and excluded from analysis.

### Data Analysis

Data from the questionnaires, attendance forms, laboratory data (HIV status), and HIV test result collection form were linked via the unique code, merged, and stored in an SPSS Statistics 22 (IBM) database. After data cleaning, statistical analysis was carried out. An analysis plan to take into account cluster sampling and a weighting factor [71] was developed using IBM SPSS Complex Samples 22. SAM who visited sites more frequently had a higher probability of selection in the study. Adjustment for this unequal selection probability was completed by calculating individual weights, based on the attendance information provided by the participant as provided on the venue attendance form. The first step of the data analysis was a univariate descriptive analysis of all outcome variables, stratified by gender, including HIV prevalence. Categorical variables were summarized by proportions and 95% confidence intervals. Nonnormally distributed quantitative data were described by median and interquartile ranges. During a second step, bivariate analysis was conducted by exploring potential determinants of HIV infection and HIV-risk taking behavior. Odds ratios were calculated to measure the association and statistical significance testing was done using a chi-square or *t* test. A multivariate analysis still has to be performed, using a logistic regression model constructed with all variables independently associated with HIV infection, sexual risk behavior, condom use, and testing behavior.

## Results

Results have been obtained from 4 interlinked studies and will be described in future research.

## Discussion

The TOGETHER Project was conceptualized to respond to the growing need for investing in targeted primary prevention interventions to reduce new HIV infections among SAM. Increasing evidence indicates that more SAM than previously assumed acquire HIV after migration to West European host countries [4-8]; however, in-depth understanding of individual, community-level, and structural factors that increase risk and vulnerability for HIV are lacking. This hampers the development and implementation of tailored responses. Applying a mix of empirically tested and innovative epidemiological and social science methods through a CBPR approach could potentially reduce this knowledge gap, while increasing community ownership, investment, and mobilization for prevention. Against this background, the project’s objective was to generate the first HIV prevalence estimates for a representative sample of SAM residing in a Western European country and to yield an in-depth understanding of the multiple factors leading to HIV infection among SAM.

Our HIV prevalence study adopted methods successfully implemented in previous bio-behavioral surveys and cross-sectional studies [30,34,35,55], that is, combining anonymous biological data from oral fluid sampling with behavioral questionnaire data collected in community venues and public areas by ethnically matched volunteer interviewers. To obtain reliable HIV prevalence estimates representative of the diverse and heterogeneous SAM communities socializing in Antwerp city, we extended these methods with a 2-stage time location sampling approach. A limitation of the time location sampling is the difficulty in estimating the probability of missing an individual who never attends any of the venues listed. We adopted extensive formative research to develop an exhaustive social map of community settings covering all aspects of social life in order to limit that probability.

Study methods and tools were continuously refined based on the input of a CAB, CRs team, and the findings of two additional formative studies. To the best of our knowledge, this was the first time that such a carefully designed CBPR design had been adopted in Europe to assess HIV prevalence among a representative sample of SAM.

Due to practical limitations, the study setting was limited to Antwerp city. While Antwerp is the major city where SAM reside in Flanders, this implies that, although the HIV prevalence study will generate a sound estimation of HIV prevalence among SAM, the results cannot be generalized to Belgium or other West European countries. Different compositions of SAM communities in other cities have to be considered. For example, in Brussels 47% of SAM are of Congolese origin [46], while in the UK a vast majority come from Eastern and Southern Africa [72].

Adopting the CBPR approach as outlined is known to be challenging in terms of assuring skills of lay researchers, and safeguarding continuous motivation and data quality [52,73]. Our experience shows that the advantages outweighed these potential limitations. Adopting a CBPR approach improved access to hidden and hard-to-reach subpopulations, ensured acceptability of the study and its methods, built community ownership for HIV prevention, respected GIPA principles, and made the HIV epidemic real to individuals in the community, as was also shown by a comparable study among MSM in Antwerp city [74]. To generate this effect, we invested in sufficient training, individual coaching, and regular meetings with the CRs and set up rigorous data quality assurance measures throughout the entire research process.

Ability to ensure community mobilization for HIV prevention may be challenged by time constraints. While some subgroups are stable communities, others are known to evolve rapidly, community venues are often unstable, and leadership fluctuates [22]. Therefore, the projects’ results concerning priority places for prevention (objective 3) need constant follow-up (eg, to track where venues moved to, to assure contacts with new leaders).

With respect to the multiple-case study, it should be mentioned that our approach to identifying factors that increase SAM’s vulnerability to HIV infection was innovative (ie, triangulation of data from life history interviews, lifelines with patient files). In particular, the use of lifelines was, according to our literature search, new to HIV research. These qualitative insights were valuable in the analysis and contextualization of the HIV prevalence study’s findings. Together, they will form a solid basis for policy recommendations and the development of future HIV prevention interventions, once all study results are available.

## Abbreviations

CAB: community advisory board
CBPR: community-based participatory research
CRs: community researchers
GIPA: greater involvement of people living with HIV/AIDS
MSM: men having sex with men
PLACE: Prioritising Local AIDS Control Efforts
SAM: sub-Saharan African migrants
TLS: time location sampling
